# Spatial and seasonal variation in disinfection byproducts concentrations in a rural public drinking water system: A case study of Martin County, Kentucky, USA

**DOI:** 10.1371/journal.pwat.0000227

**Published:** 2024-03-04

**Authors:** Jason M. Unrine, Nina McCoy, W. Jay Christian, Yogesh Gautam, Lindell Ormsbee, Wayne Sanderson, Ricki Draper, Madison Mooney, Mary Cromer, Kelly Pennell, Anna G. Hoover

**Affiliations:** 1 Department of Plant and Soil Sciences, University of Kentucky, Lexington, Kentucky, United States of America,; 2 Kentucky Water Research Institute, University of Kentucky, Lexington, Kentucky, United States of America,; 3 Martin County Concerned Citizens, Inc., Inez, Kentucky, United States of America,; 4 Department of Epidemiology and Environmental Health, University of Kentucky, Lexington, Kentucky, United States of America,; 5 Department of Civil Engineering, University of Kentucky, Lexington, Kentucky, United States of America,; 6 Department of Biosystems and Agricultural Engineering, University of Kentucky, Lexington, Kentucky, United States of America,; 7 Livelihoods Knowledge Exchange Network, Lexington, Kentucky, United States of America,; 8 Appalachian Citizens’ Law Center, Inc., Whitesburg, Kentucky, United States of America

## Abstract

To increase our understanding of the factors that influence formation of disinfection byproducts (DBPs) in rural drinking systems, we investigated the spatial and seasonal variation in trihalomethane (THM) and haloacetic acid (HAA) concentrations in relation to various chemical and physical variables in a rural public drinking water system in Martin County, Kentucky, USA. We collected drinking water samples from 97 individual homes over the course of one year and analyzed them for temperature, electrical conductivity, pH, free chlorine, total chlorine, THMs (chloroform, bromodichloromethane, dibromochloromethane, dichlorobromomethane, and bromoform) and HAAs (monochloroacetic acid, dichloroacetic acid, trichloroacetic acid, bromoacetic acid, and dibromoacetic acid). Spatial autocorrelation analysis showed only weak overall clustering for HAA concentrations and none for THMs. The relationship between modeled water age and TTHM or HAA5 concentrations varied seasonally. In contrast, there was strong variation for both HAA and THMs, with concentrations of HAA peaking in mid-summer and THMs peaking in early fall. Multiple regression analysis revealed that THM concentrations were strongly correlated with conductivity, while HAA concentrations were more strongly correlated with water temperature. Individual DBP species that only contained chlorine halogen groups were strongly correlated with temperature, while compounds containing bromine were more strongly correlated with conductivity. Further investigation revealed that increased drinking water conductivity associated with low discharge of the Tug Fork River, the source water, is highly correlated with increased concentrations of bromide. Discharge and conductivity of the Tug Fork River changed dramatically through the year contributing to a seasonal peak in bromide concentrations in the late summer and early fall and appeared to be a driver of brominated THM concentrations. Brominated DBPs tend to have higher toxicity than DBPs containing only chlorine, therefore this study provides important insight into the seasonal factors driving risk from exposure to DBPs in rural drinking water systems impacted by bromide.

## Introduction

Drinking water infrastructure is degraded and in severe need of upgrades and repairs in many parts of the United States [1]. This is particularly true in rural communities which have fewer customers per kilometer of pipe and thus large per capita infrastructure costs. Infrastructure degradation can lead to decreases in water reliability and quality, including the presence of contaminants [2]. Among the issues faced by rural public drinking water systems is formation of disinfection byproducts (DBPs) during drinking water treatment and distribution. After total coliform bacteria violations, DBP violations are the second most common violation of drinking water regulations in the United States and most violations occur in rural drinking water systems, particularly in low-income rural areas [3, 4].

Drinking water disinfection byproducts (DBPs) are formed when disinfectants (e.g. chlorine, bromine, chloramine, UV radiation, ozone) react with natural and anthropogenic organic matter and inorganic ions (I^−^ and Br^−^) during treatment of drinking water [5]. Of the many compounds that are formed, two classes of organic compounds, trihalomethanes (THMs) and haloacetic acids (HAAs), as well as the inorganic ions bromate and chlorite, are currently regulated in the United States under the Safe Drinking Water Act based on their prevalence and toxicity [6]. These compounds have been associated with a range of adverse health effects, including urinary tract cancers and adverse birth outcomes, depending on exposure level and duration [7–13], although there are many other DBP compounds of potentially higher toxicity which are unregulated [14]. Reducing concentrations of DBPs can be challenging for drinking water systems, particularly for those with degraded infrastructure and inadequate financial and technical resources, and those that rely on chlorination as a disinfection method and surface water as the source water. Further, rural drinking water systems often have large distribution networks with low demand and long service lines contributing to increased water age at the tap, which could lead to increased DBP formation [15]. Previous modeling studies have shown that smaller systems may be more susceptible to variation in DBPs due to routine variation in system hydraulic operating parameters [16]. Additionally, intrusion of contaminants into pipes that occurs during intermittent operation, as could be the case in leaky systems with frequent service outages, can lead to increased formation of DBPs [17].

Spatial variation in DBP concentrations has been well studied; however, complex interrelated factors may influence the spatial patterns of DBP formation and the composition of DBP mixtures, making it hard to generalize among water systems [18]. Changes in concentrations with distance and water age are also not necessarily linear. For example, Rodriguez et al. found that THM concentrations initially increase and then level off further from the drinking water treatment plant while HAAs initially increase with distance from the treatment plant and then decrease, presumably due to microbial degradation [19]. Villanueva et al., found that within subject variability in THM exposure over time was far greater than between subject variability [20], suggesting a limited role for spatial variability in overall exposure patterns and a greater role of seasonal variation.

Seasonal variation in DBP concentrations is often observed in drinking water systems, possibly due to factors such changes in water temperature and precursor (NOM and inorganic ion) concentrations, NOM reactivity, as well as chlorine dose [15, 19, 21–23]. As a result of large seasonal variation, quarterly samplings used in regulatory monitoring, and by extension many epidemiological studies that rely on these data, likely do not adequately capture temporal dynamics in exposure [19, 24]. While some of the health effects associated with DBPs, such as urinary tract cancers, typically require chronic exposure to develop, other effects such as cardiac birth defects have a very short window of susceptibility, even just a few weeks making short term exposures important [25]. Many authors have noted the importance of understanding seasonal variation in exposure to better understand and minimize the risk of reproductive effects of DBPs [18, 19, 21, 26].

Eastern Kentucky, the location of Martin County, has been identified as a relative hotspot for drinking water violations in the United States [3]. Prior to 2019, the drinking water system in Martin County, Kentucky had an 11-year history of violations of DBP regulations, specifically for total trihalomethanes (TTHMs; defined as the sum of the concentrations of chloroform, bromoform, chlorodibromomethane and dichlorobromomethane) and total haloacetic acids (HAA5; defined as the sum of monochloroacetic acid, dichloroacetic acid, trichloroacetic acid, bromoacetic acid, and dibromoacetic acid) [27]. For example, according to the U.S. EPA Safe Drinking Water Information System, the Martin County Water District (Public Water System ID KY0800273) received 34 maximum contaminant level violations for TTHM and HAA5. between 2006 and 2017. Thus, it can serve as a case study for investigating the formation of DBPs in rural drinking water systems.

The Martin County Water District public drinking water system was built in Martin County in the 1960s to serve around 600 households in the town of Inez, the county seat. Over the years the system was extended to serve around 4,300 households across the county without any significant upgrades to the treatment plant [27]. Because of the dissected nature of the landscape, a complex network of water lines, pumps, pressure regulating valves, and storage tanks is necessary to maintain proper pressure and flow across the service area. As much as ~69% of the produced water has been unaccounted for at service meters at times, a large proportion of which is likely due to leakage [27]. This leakage can create difficulties maintaining proper pressure and flow rates within the system, which is critical for regulating water age and possibly formation of DBPs within a distribution network [15]. For example, the leakage could make it difficult to flush the distribution lines, leading to greater biofilm formation and possibly formation of DBPs in the network due to the presence of organic matter precursors [28, 29]. Furthermore, long service lines with low demand could increase water age and potentially influence DBP formation [30].

While there have been many studies conducted on seasonal and spatial variation of DBPs in drinking water systems as highlighted above, there is limited information available about spatial and seasonal variation in small rural systems and how this potentially influences short term exposure. Given the complexity of the distribution network and the number of leaks, a key question is whether DBP exposure in certain parts of the distribution system was higher than others. It’s possible that DBP formation within the distribution network would lead to higher exposures in locations more distant from the treatment plant due to increased water age and DBP concentrations tend to increase while chlorine residuals decrease during distribution [31]. It is also possible that more complex spatial patterns could emerge given the complex network of storage tanks and pressure regulating valves present within the network coupled with the presence of long service lines with low water demand in remote areas, leading to a lack of correlation between distance from the treatment plant and water age. Sampling in this system is conducted quarterly at two locations. This small number of sampling locations and time points may fail to adequately characterize exposure in a complex network with strong seasonal variation in DBP concentrations. Further, large seasonal changes in source water chemistry due to the small size of the reservoir and river that serve as the source water may lead to large fluctuations in DBP formation.

To better characterize the spatial variation of DBP concentrations, we collected samples from 97 individual homes, randomly selected from the drinking water distribution network. We hypothesized that locations more distant from the treatment plant would have higher concentrations of THMs and HAAs based on the potential for chlorine to continue reacting with unreacted precursors present in the water or with biofilms or other deposits within the pipes. Data from the 97 individual homes were also analyzed for seasonal trends. We hypothesized that DBP concentrations would peak during the summer and reach a minimum during the winter, and that these changes would be correlated with tap water temperature, conductivity, pH, and total chlorine concentrations.

## Methods

### Ethics statement

This study was approved by the University of Kentucky Institutional Review Board (UK IRB protocol 44991). Informed consent was obtained in writing from all study participants between December 2018 and December 2019.

### Eligibility and participant enrollment procedures

We used a stratified random sampling design to select households for the study. The study population included households served by the Martin County Municipal Water District (MCMWD). Individuals who were over the age of 18, residents of Martin County, customers of the MCMWD, and could speak English were eligible to participate in the study. Some authors had access to information that could identify individual study participants during and after data collection.

**Fig 1.**
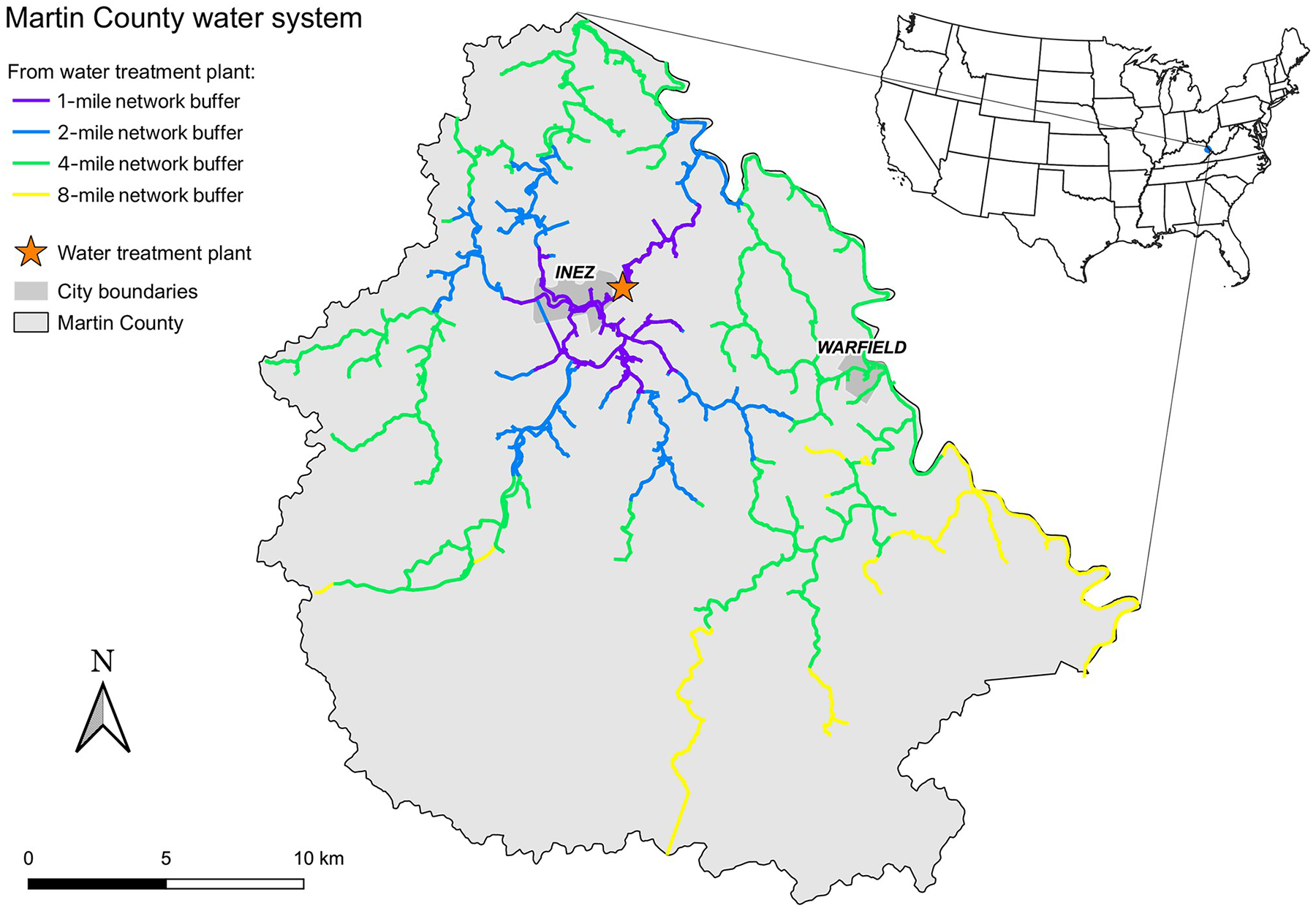
Map of Martin County, KY showing distance categories from the drinking water treatment plant that were used for distance-stratified sampling. We obtained the GIS data comprising the county boundary polygon data for Fig 1 and, thus, the base layer for these maps—from the Kentucky Geography Network (https://kygeonet.ky.gov), the spatial data clearinghouse for Kentucky. A ZIP file containing this publicly available shapefile can be downloaded from https://ky.app.box.com/v/kymartian-KyBnds-County/folder/137608414025. We used state cartographic boundary files from the U.S. Census for the inset U.S. map (https://www.census.gov/geographies/mapping-files/time-series/geo/carto-boundary-file.2015.html), and the National Hydrography Data (NHD) from the United States Geological Survey (USGS; https://www.usgs.gov/national-hydrography/national-hydrography-dataset) for the Tug Fork River.

We obtained a list of the service addresses of all customers within the MCMWD. The list was then divided into households within four discrete categories of distance from the water treatment plant (Fig 1). For each day of home visits, we focused on one distance category and rotated the distance categories. If the randomly selected resident was not home when we visited, we went to the next home on the street until we found a resident at home. We also left letters with contact information allowing the residents who were not at home to schedule an appointment at their convenience. We also collected latitude and longitude of the residence using the Global Positioning System (GPS).

### Sample collection

Sample collection was conducted according to U.S. EPA protocols for the analytical methods noted in the sample analysis section. Prior to sample collection, we opened the cold water tap until the flowing water reached a constant temperature, thus purging the premises plumbing and obtaining water from the distribution network. We collected samples for TTHM and HAA5 in pre-cleaned, pre-preserved (containing dechlorinating agent) 40 mL amber glass vials with fluoropolymer septa (TTHM) or 500 mL pre-cleaned, pre-preserved amber glass jars (HAA5). Finally, we collected samples for conductivity measurement in 50 mL polypropylene vials. We determined pH and chlorine residuals at the home as described below. All DBP samples were extracted within 14 days and extracts were analyzed within 14 days of extraction according to U.S. EPA methods. Field blanks for TTHMs and HAA5 were periodically collected and analyzed with each analytical batch. We also collected latitude and longitude of the residence using the Global Positioning System (GPS).

We collected source water samples at the Curtis Crum Reservoir and the Tug Fork River from September 2021-April 2022 to investigate the relationship between conductivity and bromide concentrations in the source water. Since we collected samples from public access boat ramps, no permission was required. Samples were collected in polypropylene vials and kept at 4°C until analysis.

### Sample analysis

Analysis of DBPs, including TTHMs and HAA5 was performed by Pace Analytical Services (Madisonville, KY, USA) by purge and trap gas chromatography mass spectrometry (TTHMs) or derivatization and gas chromatography with electron capture detection (HAA5), following U.S. EPA methods 524.2 revision 4.1[32] and 552.2 revision 1 [33], respectively. Pace Analytical is accredited by the Kentucky Department of Environmental Protection for analysis of drinking water samples for regulatory compliance.

We determined conductivity using a 5-ring conductivity cell with integrated temperature sensor and conductivity module (model 865, Metrohm, Herisau, Switzerland), which was calibrated to a certified reference conductivity standard.

We determined pH, total chlorine, and free chlorine content during the home visits. We determined pH using phenol red as a pH indicator and a hand-held colorimeter (model DR300, Hach, Loveland, CO, USA). We determined free and total chlorine concentrations using the N,N-diethyl-p-phenylenediamine (DPD) method and the Hach DR300 pocket colorimeter (U.S. EPA method 330.5) [34]. Accuracy of the free and total chlorine method was verified by analyzing ultra-pure water as a blank and certified reference materials for chlorine residuals (Inorganic ventures, Christiansburg, VA, USA).

We analyzed surface water samples for bromide using a ion chromatography coupled to inductively coupled plasma mass spectrometry (IC-ICP-MS) using previously described methods [35]. Samples were analyzed using an Agilent 1200 series chromatography system using a Dionex AS-11HC column. An Agilent 7900 ICP-MS was used to quantify bromide using m/z = 80. NIST traceable standards were obtained from Inorganic Ventures (Christiansburg, VA, USA).

### External data sources

We obtained data on discharge of the Tug Fork River from the U.S. Geologic Survey (USGS), National Water Information System from December 15, 2018-January 15, 2020 from USGS station 03213700 at Williamson, WV (https://waterdata.usgs.gov/nwis). Air temperature and precipitation data were obtained over the same period from the National Climate Data Center for the Inez, KY, USA, Global Historical Climatology Network ID USC00154138 (https://www.ncdc.noaa.gov). Data for regulatory monitoring samples for DBPs were obtained from the Kentucky Division of Water (https://dep.gateway.ky.gov/DWW/).

### Data analysis and mapping

We performed multiple linear regression analyses, pearsons correlations, linear regression and calculated descriptive statistics using SPSS version 26 (IBM, Armonk, NY) or with the R statistical package and generating plots with package ggplot2 (http://had.co.nz/ggplot2/book). For multiple linear regression analyses we used backward model selection and initially entered water temperature, free chlorine, total chlorine, conductivity, pH, network distance from and water age. The selection criterion for removal was p > 0.10. Regression was performed separately for TTHM, HAA5, and each of the component species. Additionally, we divided the distribution network into six distinct branches or sections based on water consumption zones (S1 Fig) and performed multiple regression analysis separately for each section individually as well as a combined analysis after coding section as a dummy variable. In all statistical analyses the level of statistical significance was considered to α = 0.05.

We calculated the global Moran’s I in GeoDa (https://geodacenter.github.io) software to assess spatial autocorrelation, or spatial clustering of similar values, for TTHMs and HAA5, using spatial weights based on each participating household’s four nearest neighbors. The global Moran’s I ranges from −1 to 1, with a positive I indicating clustering of similar values, and negative I indicating dispersion of similar values. This analysis thus reveals whether there is a geographic pattern in contaminant concentrations, which would be expected if DBP concentrations increased with distance from the water treatment plant. We analyzed all TTHM and HAA5 values regardless of season, as well as values for each season separately, though there were too few Spring samples (n = 6) for meaningful analysis.

For mapping, we obtained GIS shapefiles comprising the county boundary polygons from the Kentucky Geography Network (https://kygeonet.ky.gov), the spatial data clearinghouse for Kentucky. A ZIP file containing this publicly available shapefile can be downloaded from https://ky.app.box.com/v/kymartian-KyBnds-County/folder/137608414025. We used state cartographic boundary files from the U.S. Census for an inset U.S. map (https://www.census.gov/geographies/mapping-files/time-series/geo/carto-boundary-file.2015.html), and the National Hydrography Data (NHD) from the United States Geological Survey (USGS; https://www.usgs.gov/national-hydrography/national-hydrography-dataset) to add the Tug Fork River.

Physical and topological data used for use in building a computer model of the Martin County water distribution system was obtained from the Kentucky Water Resource Info System (WRIS) at https://kia.ky.gov/WRIS/Pages/WRIS-Portal.aspx. This information was verified in the field and adjusted when necessary after consultation with officials with the Martin County Water District (MCWD). Additional information on the spatial and temporal distribution of water demands, including estimated water loss, were also obtained from MCWD officials.

A commercial software package for modeling water distribution hydraulics (KYPIPE) was used to estimate water age [36]. KYPIPE calculates water age using algorithms originally proposed and used in EPANET [37] a similar software package for use in modeling water quality parameters. EPANET calculates water age at various points in the distribution system using a Lagrangian time-based approach to track discrete parcels of water as they move through the distribution system. The average expected water age at any point in the system can then be approximated by running the model over an extended number of days until the water ages reach an equilibrium value. This is necessary to balance out the contributions of water ages from different sources to the system (e.g. water storage tanks). In the current study, the model for the Martin County system was run for 200 hours to establish equilibrium and then water ages associated with each sample point were estimated based on their proximity to the nearest node in the model. To ensure the accuracy of the model estimates, the hydraulic parameters of the model (i.e., pipe roughness and spatial and temporal water demand) were first calibrated to match observed flows and pressures collected from fire hydrant flow tests in the field. The accuracy of the spatial demands was improved by partitioning the model into 23 water consumption zones (S1 Fig) which were separated by master water meters. Demands within each zone were then obtained by performing a mass balance of each zone. Estimates of water loss were then calculated by subtracting the sum of the individual customer meter readings within a zone from the total estimated demand for that zone. This process helped ensure a more accurate distribution of demands and helped minimize the impact of the aggregate water loss across the system. Field measurements of chlorine residuals and THMs were then to validate the relative water ages for the final model.

## Results

### Disinfection byproducts

Descriptive statistics for TTHMs and HAA5 concentrations are summarized in Table 1. Mean concentrations of TTHMs (0.065 mg/L) and HAA5 (0.035 mg/L) were below the U.S. EPA maximum contaminant levels (MCLs) of 0.08 and 0.06 mg/L, respectively. We measured concentrations in 28 individual samples that exceeded the EPA MCL for TTHMs (0.08 mg/L) and 10 that exceeded the MCL for HAA5 (0.06 mg/L). Since our observations are heavily weighted towards the summer and fall, our mean concentrations may differ from the true annual mean concentrations. Average detected concentrations for individual DBP compounds as compared to the MCLs and maximum contaminant level goals (MCLGs) are presented in Table 2.

### Temperature, pH, conductivity, and chlorine residuals

Table 1 shows the descriptive statistics for chlorine residuals, pH, conductivity, temperature, and total dissolved solids estimated from conductivity measurements. Generally, these values were within the normal expected range for potable water, except for a few isolated observations of inadequate chlorine residuals during the summer (<0.2 mg/L free chlorine).

### Spatial variation and water age

**Fig 2.**
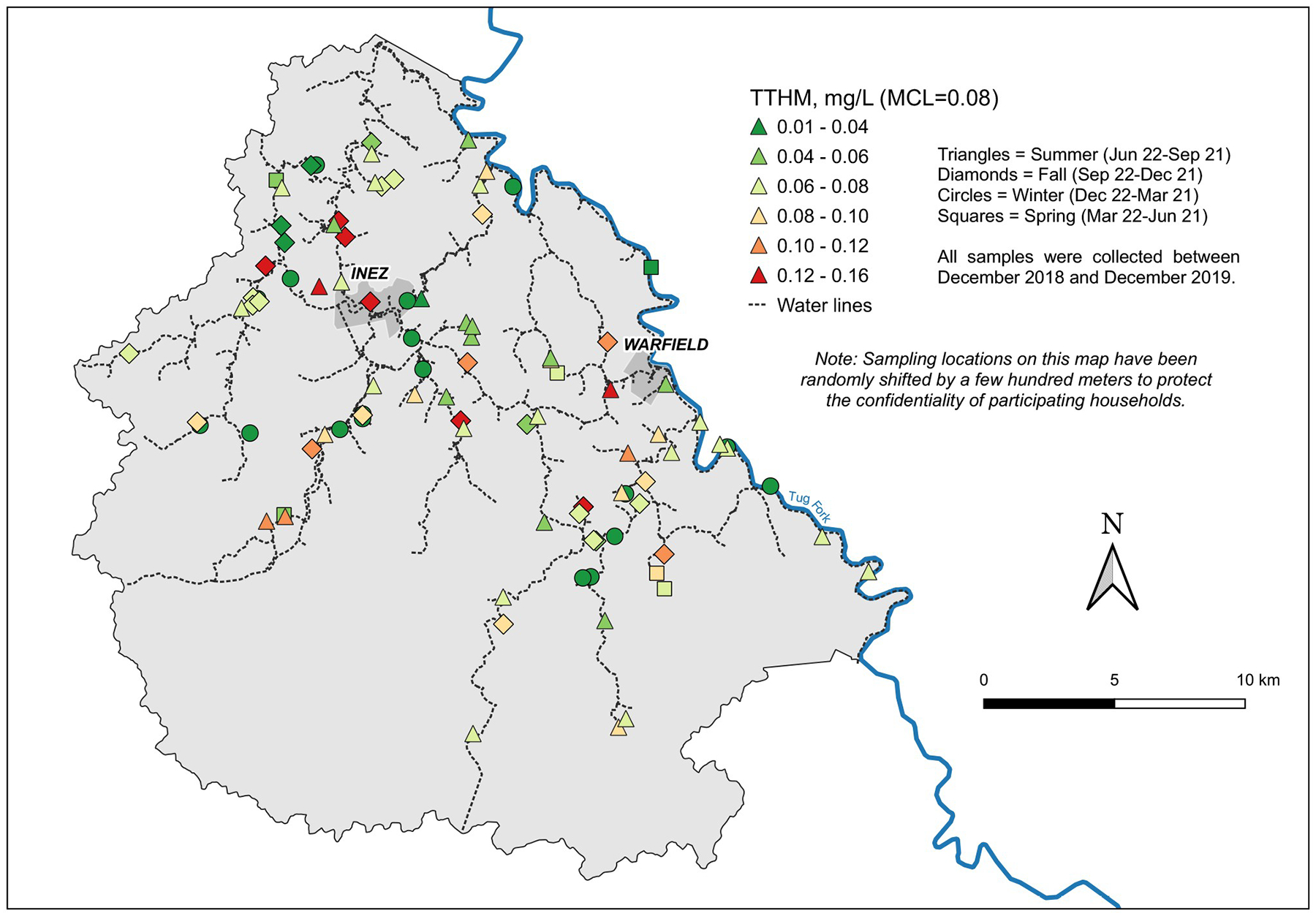
Distribution of total trihalomethane (TTHM) concentrations in Martin County, Kentucky from Winter 2018-Winter 2019. We obtained the GIS data comprising the county boundary polygon data for Fig 2 and, thus, the base layer for these maps—from the Kentucky Geography Network (https://kygeonet.ky.gov), the spatial data clearinghouse for Kentucky. A ZIP file containing this publicly available shapefile can be downloaded from https://ky.app.box.com/v/kymartian-KyBnds-County/folder/137608414025. We used state cartographic boundary files from the U.S. Census for the inset U.S. map (https://www.census.gov/geographies/mapping-files/time-series/geo/carto-boundary-file.2015.html), and the National Hydrography Data (NHD) from the United States Geological Survey (USGS; https://www.usgs.gov/national-hydrography/national-hydrography-dataset) for the Tug Fork River.

**Fig 3.**
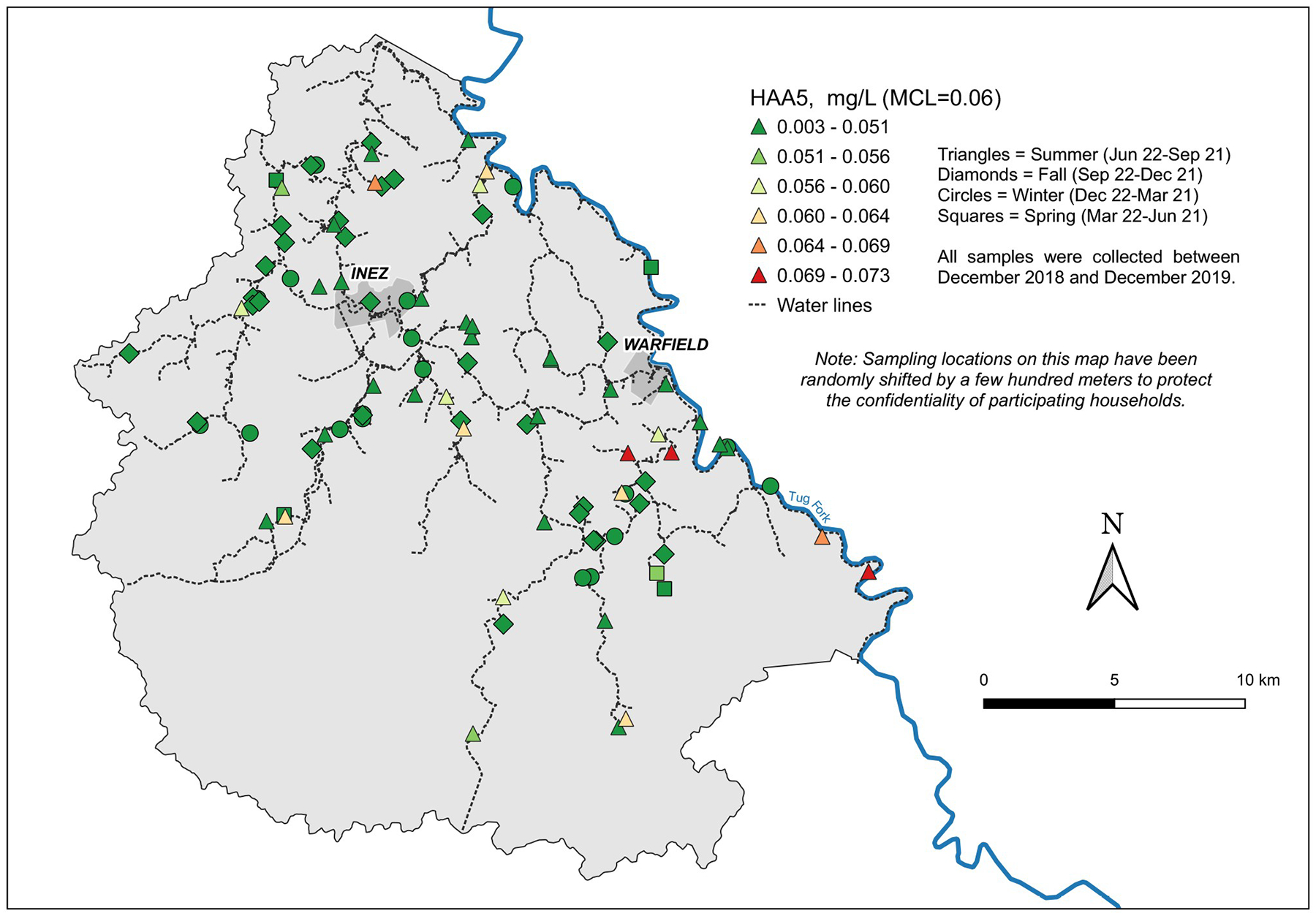
Distribution of total haloacetic acid concentrations (HAA5) in Martin County, Kentucky from Winter 2018-Winter 2019. We obtained the GIS data comprising the county boundary polygon data for Fig 3 and, thus, the base layer for these maps—from the Kentucky Geography Network (https://kygeonet.ky.gov), the spatial data clearinghouse for Kentucky. A ZIP file containing this publicly available shapefile can be downloaded from https://ky.app.box.com/v/kymartian-KyBnds-County/folder/137608414025. We used state cartographic boundary files from the U.S. Census for the inset U.S. map (https://www.census.gov/geographies/mapping-files/time-series/geo/carto-boundary-file.2015.html), and the National Hydrography Data (NHD) from the United States Geological Survey (USGS; https://www.usgs.gov/national-hydrography/national-hydrography-dataset) for the Tug Fork River.

Spatial autocorrelation analysis revealed significant, but weak, clustering of similar concentration values during the summer, but not in the winter or fall, for TTHMs (Table 3). Spatial autocorrelation analysis revealed stronger spatial clustering across all seasons for HAA5 than for TTHMs (Table 3). Note that we did not have enough samples in the spring for spatial autocorrelation analysis. Identification of local hot spots from the map for TTHM concentrations (Fig 2) is difficult. Although in the fall, there appeared to be a group of samples near the treatment plant with high TTHM concentrations, there was no statistically significant clustering of values during that period. For HAA5, the highest concentrations tended to occur at locations that were further from the treatment plant, although there were also observations with low HAA5 concentrations distant from the treatment plant, even during the summer (Fig 3). Multiple regression analysis revealed a significant positive effect of distance through the pipe network from the treatment plant on HAA5 concentrations when including physicochemical properties of the water in the model that were significant (conductivity, pH, temperature) ((*F*_*3,92*_ = 23.08, *p <0.001*, *R*^*2*^ = 0.57; standardized (std) β for distance = 0.298, *p*<0.000; S1 Table) but no effect on TTHM concentrations (p = 0.916 for distance, *F*_*3,92*_ = 135.3, *p* <*0.001*, *R*^*2*^ = 0.82; S2 Table). Multiple regression analyses conducted for both TTHM and HAA5 using the six sections defined in S1 Fig revealed that section had no significant effect (p values ranging from 0.395–0.977) and was thus dropped from the models during backward selection. Models run on sections individually also revealed no change in the overall conclusions although this approach resulted in reduced statistical power in the individual models as opposed to a model including all sampling locations. For example, section 5 did not have enough degrees of freedom to run the full model. Distance and water age were not significant for any section for TTHMs and were significant for sections 2 and 3 for HAA5.

Further examination of the individual species of HAA5 that had most observations above the method detection limit (dibromoacetic acid (DBA), dichloroacetic acid (DCA), and trichloroacetic acid (TCA)), showed statistically significant positive relationships between concentrations and distance for DCA and TCA but not DBA. The std β values for DCA and TCA were 0.171 (*p* = 0.045) and 0.165 (*p* < 0.022), respectively (See S3–S5 Tables).

Overall, water age modeled using KYPIPE was not significantly correlated with distance from the water treatment plant (*r* = 0.174, *p* = 0.115). This stems from the complexity of the distribution network which includes many long service lines with low demand as well as multiple branches with differing water demands. Overall, water age had no statistically significant relationship with TTHM or HAA5; however, when examining this relationship month by month, there was a marginally significant relationship between water age and TTHM concentrations in January 2019 (S2 Fig; *p<0.06*, *r*^*2*^ = *0.23*), and December 2019 (S2 Fig; *p = 0.07*, *r*^2^ = 0.51). The relationship between estimated water age and HAA5 was marginally significant for July 2019 (S3 Fig; *p = 0.06*, *r*^2^ = 0.15) and significant for August 2019 (S3 Fig; *p = 0.01*, *r*^2^ = 0.74).

### Temporal variation and relationship to water chemistry and temperature

**Fig 4.**
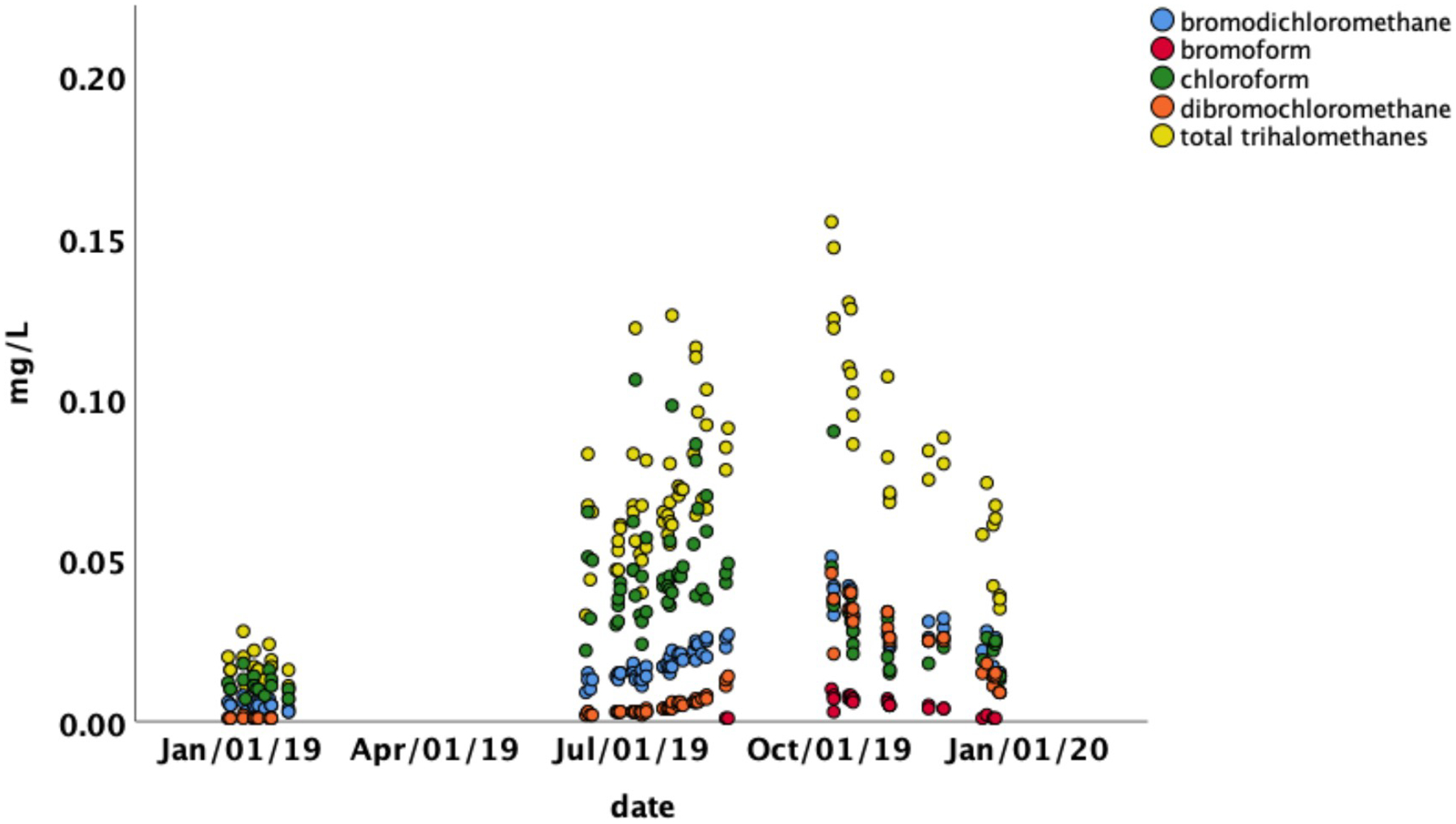
Total trihalomethane concentrations in drinking water from households in Martin County Kentucky as a function of date. Lack of a data point for a date indicates that the concentration was below the method detection limit.

**Fig 5.**
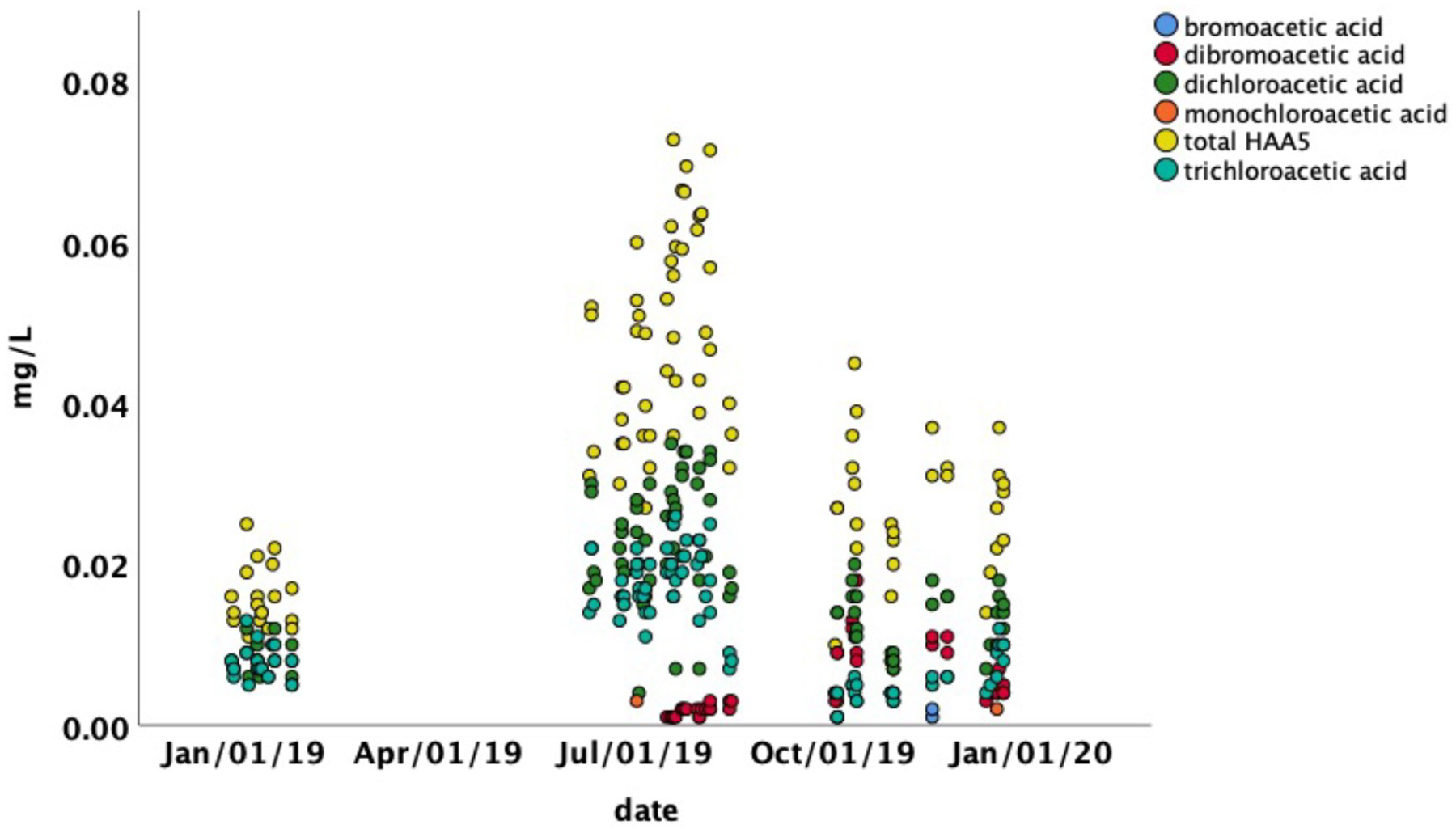
Haloacetic acid concentrations in drinking water from households in Martin County Kentucky as a function of date. Lack of a data point for a date indicates that the concentration was below the method detection limit.

Visual inspection of the temporal distribution of TTHMs and HAA5 (Figs 4 and 5; Table 1) suggested significant seasonal variation in concentrations. The highest values for TTHMs and HAA5 in individual samples occurred exclusively in the summer and fall from June to November. There were differences in the temporal variation between TTHMs and HAA5 and among individual chemical species within those two classes. These differences were analyzed by relating them to physicochemical properties of the tap water.

**Fig 6.**
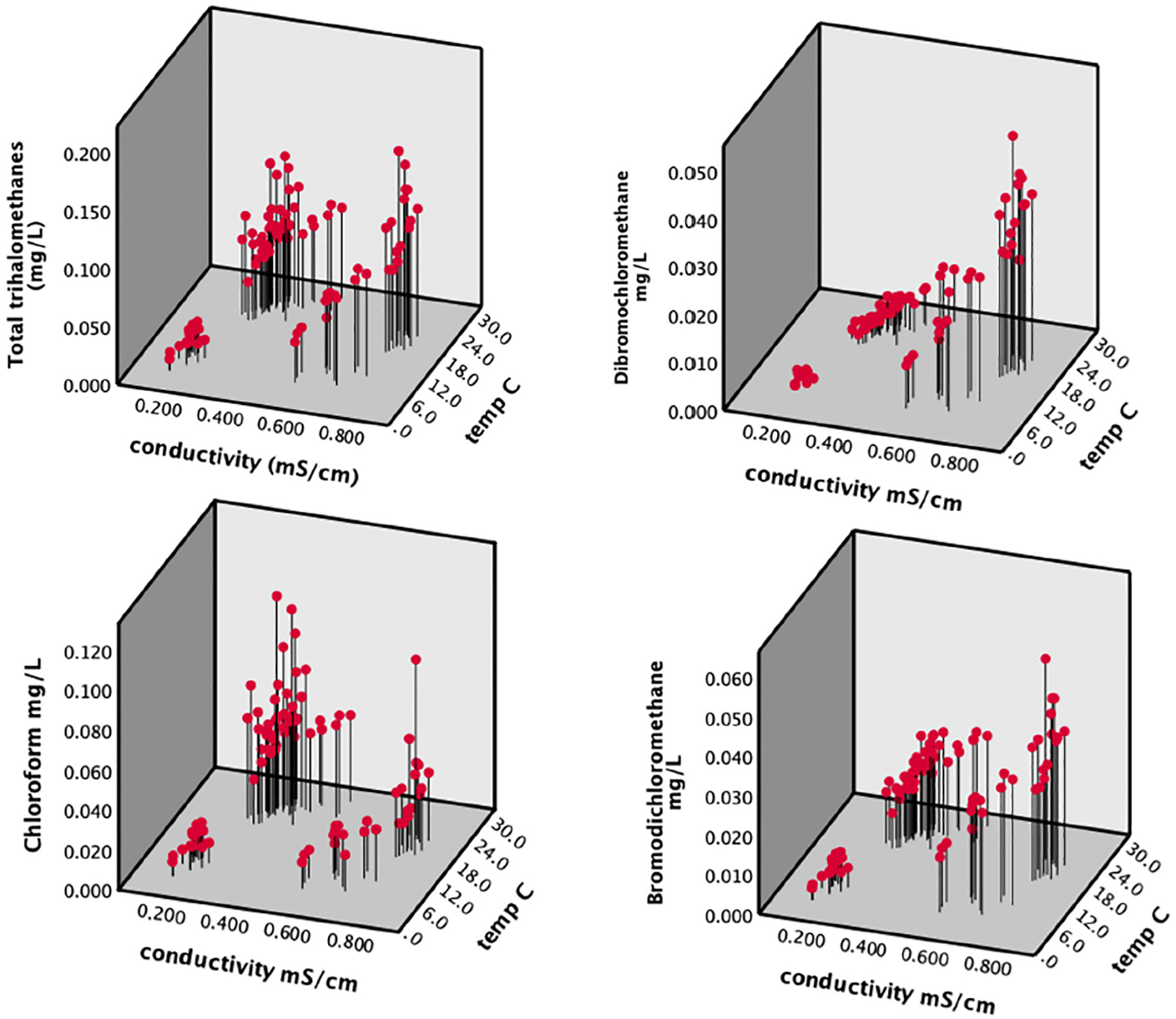
Total trihalomethane (TTHM) concentrations as a function of tap water conductivity and temperature (top) along with concentrations of individual THM species (bottom).

Multiple regression analysis (S2 Table) revealed that free chlorine, temperature, and conductivity were significantly associated with TTHMs but distance and pH were not. Overall, the model was highly significant and explanatory of TTHM concentrations (*F*_*3,92*_ = 135.3, *p <0.001*, *R*^*2*^ = 0.82). Conductivity and temperature had a positive correlation with TTHMs and total chlorine had a negative correlation (S2 Table). Substituting modeled water age for distance did not change the *R*^2^ value and water age was not significant (not shown). Concentrations peaked in late summer and early fall. The relationship between TTHM concentrations, temperature and conductivity are shown in Fig 6. Peak conductivity of drinking water occurred at the minimum discharge of the Tug Fork River (S4 Fig). This coincided with the peak in TTHM concentrations (Fig 4), and a period of drought during the month of September 2019 (S4 and S5 Figs). The peak water temperature coincided with peak mean observed air temperature in July (S5 Fig). Tap water temperature was strongly correlated with air temperature (Pearson’s correlation; *r* = 0.901, *p*<0.001).

Further regression analysis on individual THM compounds detected in individual samples showed a pattern dependent upon the halogen groups present. As the degree of bromine substitution (i.e. number of bromine atoms per molecule) for a compound increased, conductivity became more strongly correlated with its concentrations. Concentrations of chloroform were more strongly related to water temperature, while compounds containing bromine (bromodichloromethane and dibromochloromethane) were more strongly related to conductivity (Fig 6; S2–S4 Tables). For example, conductivity was not significant for the multiple regression model with only temperature (std β = 0.621) and free chlorine (std β = −0.362) being significant (S6 Table). Conversely, the std β for dibromochloromethane for conductivity was 1.084 and the std β for temperature was −0.069 (S7 Table). For bromodichloromethane, the std β values were 0.710 and 0.406 for conductivity and temperature, respectively (S8 Table). Bromoform was not detected in most of the samples, so we did not perform multiple regression for this species; however, except for one sample, it was only detected in the fall when conductivity was at its highest.

In contrast, the peak concentrations of HAA5 (Fig 5) coincided with the peak air temperature observed in Martin County (S5 Fig). It was during this period that high concentrations of HAA5 occurred in individual samples, primarily in locations remote from the treatment plant (Fig 3). Concentrations of HAA5 showed temporal variation that differed from TTHMs, with the peak concentrations occurring in the summer rather than the early fall (Fig 5). Multiple regression analysis revealed that the most important factors associated with HAA5 were temperature, conductivity, and distance. The pH was not statistically significant (S1 Table). The absolute value of the coefficient for temperature was more than double the absolute values of the other coefficients. It is notable that the coefficient for conductivity for HAA5 was negative while it was positive for TTHMs. Overall, the model for HAA5 was less predictive than the TTHM model, but still statistically significant (*F*_*3,92*_ = 23.08, *p <0.001*, *R*^*2*^ = 0.57). When modeled water age was substituted for distance, water age was not significant (in contrast to distance which was), but the overall *R*^2^ value was unchanged.

**Fig 7.**
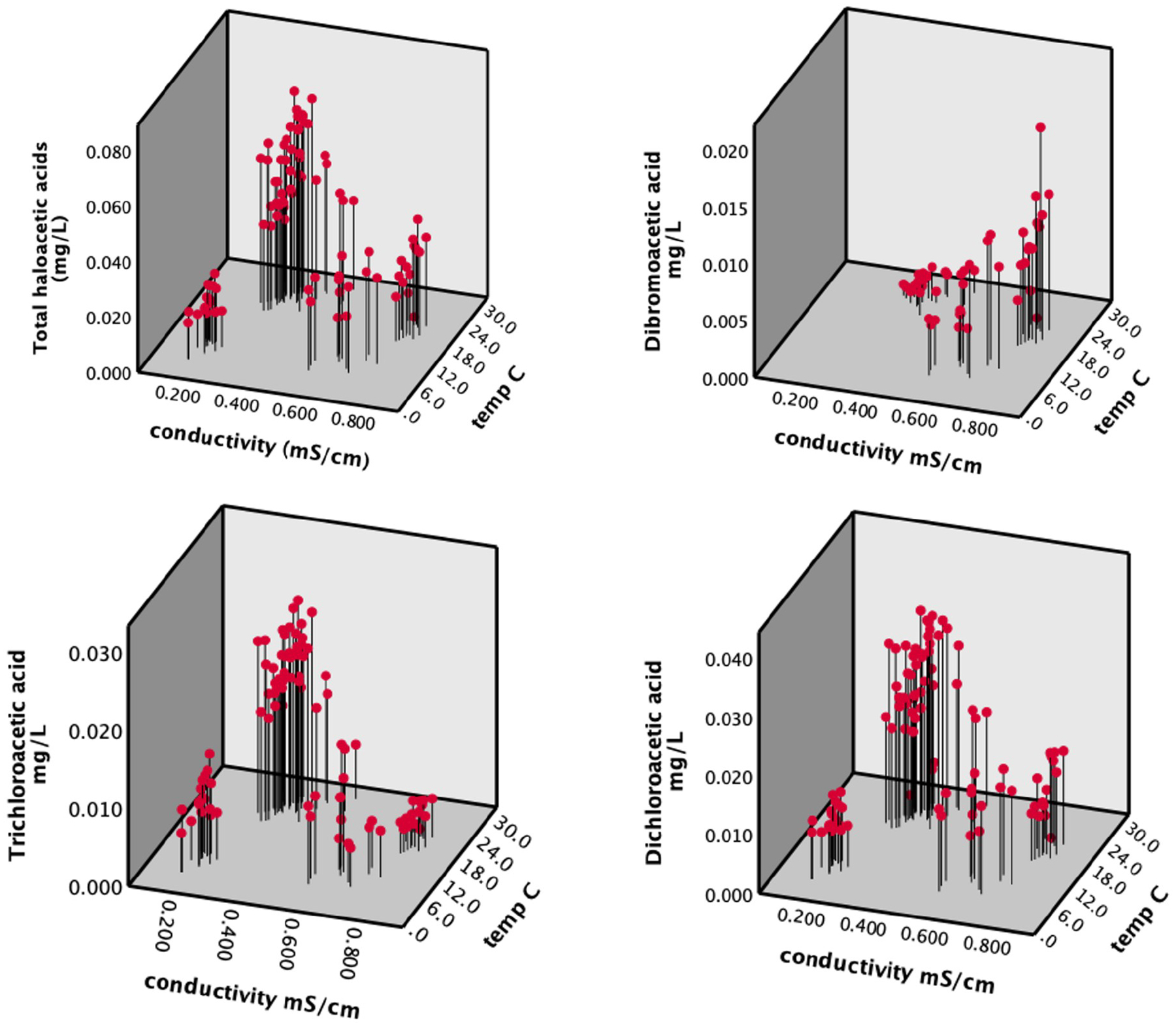
Total haloacetic acid (HAA5) concentrations as a function of tap water conductivity and temperature (top) along with concentrations of individual haloacetic acid species (bottom).

When analyzing individual species of HAA5 with a significant number of observations above the method detection limit (dibromoacetic acid (DBA), dichloroacetic acid (DCA), and trichloroacetic acid (TCA)), we observed that temperature had a stronger relationship with concentrations than conductivity on the chlorinated compounds (DCA and TCA), and conductivity while the opposite was true for DBA (S3–S5 Tables Fig 7). The std β value for conductivity for DBA was 0.850 while temperature was insignificant. For DCA and TCA, there was a positive effect of temperature (β values of 0.652 and 0.672) and a negative effect of conductivity (β values of −0.577 and −0.956). This again showed that chlorine substitution increased with temperature and bromine substitution increased with conductivity. Because monochloroacetic acid was only detected in two samples and bromoacetic acid was only detected in six samples, they were not included in our multiple regression analysis.

To follow up on the correlation between conductivity and increasing concentrations of brominated trihalomethane and haloacetic acid concentrations, we collected samples from the Tug Fork River and the Crum reservoir between September 2021 and May 2022 (S6 Fig). There was a significant positive relationship between conductivity and bromide concentrations in both bodies of water (r^2^ = 0.77 and 0.95, respectively).

## Discussion

This study adds to our understanding of seasonal and spatial variation of DBPs in small rural drinking water systems in temperate climates. While this study was initially designed primarily to capture spatial variation within the system by visiting many locations, the data analysis revealed strong seasonal variation which appeared to be the source of much greater variation. Thus, in addition to average concentrations, we investigated whether there was significant variation in DBP concentrations throughout the drinking water distribution network, in time, and in relation to select water properties.

We found widespread occurrence of TTHMs and HAA5, with concentrations for HAA5 peaking in the middle of the summer and TTHM concentrations peaking in the fall. The seasonal patterns observed here are similar to those found in previous studies. For example, Rodriguez et al. also observed higher concentrations of THMs in the summer and fall; however, HAA concentrations were highest in the spring as opposed to the summer in the present study [19]. After sampling in a single location repeatedly for a year, Wang et al., found that both THMs and HAAs had their highest concentrations in the summer; however, there seemed to be less variation in brominated THMs than in our study, perhaps indicating less seasonal change in bromide concentrations [38]. Baytak et al., observed a different pattern than most studies where DBP concentrations were at their highest in the winter [21]. They attributed this to higher non-purgeable organic carbon (NPOC) concentrations observed in surface source water winter relative to other seasons. It is also important to note that this study was conducted in Turkey, which has a Mediterranean climate, contrasting with the humid subtropical climate of Kentucky, so there are large differences in rainfall patterns.

Some previous studies have observed spatial patterns of TTHM concentrations that differ from what we observed. For example, research on a system in Cyprus found that household TTHM levels increase with increasing distance from the chlorination point [15]. Increased residence time has also been shown to be positively correlated with TTHM concentrations [15, 39]. These conclusions are consistent with our HAA5 findings, but differ from our TTHM findings, where distance from the chlorination point was not a key factor overall. Since water age, rather than the distance travelled, is likely to be a better predictor of DBP concentrations, we also investigated the relationship between modeled water age and DBP concentrations and only found significant relationships during certain months. While it is known that both TTHM and HAA5 concentrations increase with water age, it is likely that the strong temporal variation in our data set make it difficult to discern this pattern as the temporal variation appears to be much greater than the variation due to water age. For TTHMs, water age was significantly related to DBP concentrations only during the winter months when seasonal factors (changes in temperature and conductivity) were changing more slowly than during the summer months. It has been observed previously that spatial variation in DBP is not temporally consistent [18]. Rodriguez et al found water residence time, as measured by fluoride tracer, to be an important factor determining DBP concentrations [19]. However, our system had longer predicted residence times which could be as long as 200 hours compared to only up to 36 hours in the Rodriguez et al. In future studies in small systems with such large temporal variation, it is probably necessary to sample at many locations simultaneously or within a very short time to characterize the water age effect.

Previous work has also demonstrated seasonal variation is closely linked with water temperature, organic matter concentrations, and organic matter reactivity [15, 21–23, 40, 41]. Often, drinking water utilities use higher doses of chlorine during the summer when microbial loads are higher [5]; however, we did not observe a positive correlation between total chlorine and DBP concentrations. Our finding that conductivity is correlated with brominated THM and HAA concentrations is explained by the correlation between conductivity and bromide concentrations in source water in our follow-up sampling. Previous studies of effects of seasonal changes in source water hydrology and watershed process on DBP precursor concentrations have largely focused on dissolved organic matter rather than bromide [14, 42–44]. Chlorination of bromide-containing waters forms HOBr, which is more reactive to natural organic matter than HOCl by an order of magnitude resulting formation of brominated DBPs [45]. Previous studies have observed that hydrophilic organic matter of lower molecular weight is more susceptible to bromination, while more aromatic NOM of higher molecular weight is more susceptible to chlorination [46], which could also contribute to seasonal changes in bromine substitution. Correlation between bromide concentrations and high conductivity has been observed in periods of low discharge of the Allegheny river in Pennsylvania, USA, in a stretch of the river receiving effluent from treated oil- and gas-produced water discharges [47]. This river system is also located within the Appalachian Mountain region of the USA. Indeed, the majority of predictive models for THM formation include the Br^−^ concentration as a key term [5]. It is important to note that our study did not investigate all the brominated HAAs. In addition to dibromoacetic acid, there are four unregulated brominated HAAs. Future studies should include these compounds to determine if the trends observed between bromide concentration and degree of bromine substitution are consistent when all brominated HAAs are included.

Formation of DBPs is also known to increase at higher temperatures during chlorination [48]. increased reaction rates simply due to temperature is a simple explanation; however, seasonal changes that are important and are merely correlated with increased temperature, such as in the concentrations and reactivity of the NOM cannot be ruled out as a contributing factor [41]. It is also important to note that although we found a significant effect of pH on HAA5 and not THMs, the pH value of the finished drinking water may differ from the pH during disinfection. Previous studies have shown that DBP formation is influenced by pH during water treatment [46, 49]. Note that the pH we measured at the tap may differ from the pH during the water treatment process. Unfortunately, sampling of source water contemporaneously with the tap water sampling was beyond the scope of this study, so it is left to future studies to examine the relationship between source water characteristics and finished tap water characteristics in detail.

Understanding the factors that determine the relative formation of brominated and chlorinated DBPs is a priority given the higher toxicity of brominated compounds in general. Bromine is ubiquitous in sea water; however, concentrations in inland water are general low [50]. Potential sources of Br- in the region include natural weathering of the abundant organic-rich shales and coal, as well as accelerated release of Br from these materials from coal mining activity or fracking [51]. Much of the land area of Martin County has been surface mined for coal. Coal-combustion waste from power plants is another potential source [52]. In addition to the natural presence of Br in coal, Br is added to coal, or brominated activated carbon is used to help in the removal of Hg from flue gasses in the United States [53]. Bromine is present within the coal combustion wastes and is elevated far above background in coal-fired power plant wastewaters and can be as high as bromide concentrations in sea water, concentrations of bromide in fracking produced waters can be even higher [54]. Concerns have been raised about formation of brominated DBPs in drinking water utilities which are downstream from coal-fired power plants in recent years [52, 55]. The present study also raises the question of the impact of periods of drought or low flow conditions in source water on brominated DBP concentrations particularly since increased frequency and severity of drought is expected in the southeastern United States due to climate change in the coming decades [56].

The highest concentrations of both THMs and HAA5 observed in this study were more than double the annual averages (0.155 vs 0.065 mg/L for TTHM; 0.073 vs 0.035 mg/L for HAA5). The LRAA based on the regulatory monitoring samples collected at the Meathouse Road pumping station, which typically has the highest values of the two regulatory monitoring stations, was only 0.056 and 0.040 mg/L for TTHMs and HAA5, respectively, during the quarter in which we observed peak TTHM and HAA5 concentrations (https://dep.gateway.ky.gov/DWW/). Mean concentrations of TTHMs in Fall were more than triple the mean concentrations in winter (0.099 vs 0.030 mg/L). Mean concentrations of HAA5 were more than double in summer compared to the winter (0.020 vs 0.049 mg/L). This is similar to what was observed by Rodriguez et al., who observed TTHM concentrations averaged five times higher in summer and fall than in winter and HAA concentrations averaged four times higher in spring than winter [19]. While long-term exposures relevant to chronic health effects such as cancer may be well characterized by long term averages, such as the LRAA, the utility of the LRAA in characterizing short-term fluctuations in exposures, as observed in this study, and their impact on non-cancer endpoints such as birth defects warrants further study. For example, health effects which have been linked to THMs, particularly brominated THMs, include cardiac birth defects [9]. The heart has a critical window of susceptibility to teratogenesis during development within the first trimester. For example, conotruncal defects, which are associated with THMs, have a window of susceptibility between the 3^rd^ and 7^th^ weeks post-conception [9, 25]. Previous studies have also demonstrated that short-term exposures typically exceed the LRAA [26]. However, because most epidemiological studies rely on quarterly exposure data of TTHM and HAA5 from regulatory data, the relationship between short-term exposures and adverse birth outcomes is not well characterized [9, 26, 57].

The average concentrations of DBPs observed in this study did not exceed the U.S EPA MCLs; however, several MCLGs were exceeded. The EPA MCLs consider multi-route exposure from drinking water (ingestion, dermal, inhalation) as well as exposure from other sources. Several considerations including animal studies, epidemiological data, economic feasibility, and economic analyses are used to determine the MCLs. The MCLs are a balance between economic and technical considerations and the likelihood of adverse health outcomes. The maximum contaminant level goals are the concentrations at which adverse health effects are not expected to occur and are the goals for protection of public health. Concentrations in our study exceeded these MCLGs for several compounds, particularly the more toxic brominated compounds (Table 2). Based on the U.S. EPA MCLGs, bromodichloromethane, bromoform, and dichloroacetic acid were the main drivers of risk of the measured compounds if only the individual effects of the compounds are considered. It is important to note that hundreds of DBPs have been identified and that THMs and HAAs are not necessarily a good indicator of exposure to potentially more toxic DBP compounds which are present [58, 59]. Other sources of DBPs exposure such as swimming in chlorinated water are also important [20].

## Conclusions

This study identified factors that are associated with DPB concentrations in a small rural drinking water system and demonstrated that dry summer conditions where source waters are at low flow conditions, increases in more toxic brominated DBPs are expected. It also demonstrated that spatial variation is complex and is season and DBP species-specific. This adds to the body of knowledge on sources of spatial seasonal variation in DBP concentrations in small rural drinking water systems in temperate climates. While this study is limited in that only one year of data were collected, there were gaps in sampling of the time series, and limited information on source water properties was obtained, it does provide important insights that can inform future research. Factors in small drinking water systems such as reliance on small rivers and streams and small reservoirs could exacerbate fluctuations in water temperature chemistry during periods of drought, which was related to increased DBP formation. The presence of long service lines with low demand also likely contribute to DBP formation. Future efforts at reducing DBP exposure could address seasonal changes in source water chemistry and how adjustments to operations might be made to reduce the formation of DBP compounds. Some measures, particularly those related to source water quality, might be system specific. In the case of Martin County, pumping from the reservoir during the spring and winter months and avoiding pumping from the river during periods of low river discharge during the late summer and early fall could reduce bromide concentrations in the source water which could then lead to a decrease in brominated DBP formation. Increasing the capacity of the reservoir could also lessen the need to pump water from the river during the late summer and early fall. Other measures might be more generalizable. For example, the utility could consider conducting more frequent (both temporal and spatial) sampling during the more critical summer months to monitor and detect increased DBP concentrations and then conduct targeted main flushing in those segments. Altered storage tank management during the summer could also be considered.

## Supplementary Material

S1 S1 Table. Multiple regression coefficients for haloacetic acids (HAA5).

S3 S3 Table. Multiple regression coefficients for dichloroacetic acid.

S2 S2 Table. Multiple regression coefficients for total triahalomethanes.

S4 S4 Table. Multiple regression coefficients for trichloroacetic acid.

S5 S5 Table. Multiple regression coefficients for dibromoacetic acid.

S6 S6 Table. Multiple regression coefficients for chloroform.

S7 S7 Table. Multiple regression coefficients for dibromochloromethane.

S8 S8 Table. Multiple regression coefficients for bromodichloromethane.

S9 S1 Fig. Water consumption zones used for multiple regression analysis for the Martin County Water District labelled using different colors as indicated in the key.

S10 **S2 Fig. Total trihalomethanes plotted as a function of estimated maximum water age by month.** Solid line is the least squares fit of the data and the shaded areas represent the 95% confidence interval for the fitted model. We obtained the GIS data comprising the county boundary polygon data for Fig 3 and, thus, the base layer for these maps—from the Kentucky Geography Network (https://kygeonet.ky.gov), the spatial data clearinghouse for Kentucky. A ZIP file containing this publicly available shapefile can be downloaded from https://ky.app.box.com/v/kymartian-KyBndsCounty/folder/137608414025. We used state cartographic boundary files from the U.S. Census for the inset U.S. map (https://www.census.gov/geographies/mapping-files/timeseries/geo/carto-boundary-file.2015.html), and the National Hydrography Data (NHD) from the United States Geological Survey (USGS; https://www.usgs.gov/national-hydrography/national-hydrography-dataset) for the Tug Fork River.

S11 **S3 Fig. Total haloacetic acids (HAA5) plotted as a function of estimated maximum water age by month.** Solid line is the least squares fit of the data and the shaded areas represent the 95% confidence interval for the fitted model.

S12 S4 Fig. Relationship between discharge of the Tug Fork River at Williamson, WV and conductivity of drinking water in Martin County, KY.

S13 S5 Fig. Precipitation and observed air temperature at Martin County, KY from December 2018 to January 2020.

S14 **S6 Fig. Relationship between conductivity and bromide concentrations in the Tug Fork River at Kermit West Virginia, USA and the Curtis Crum Reservoir, Martin County, KY USA.** Samples collected between September 2021 and May 2022.

## Figures and Tables

**Table 1.** Temperature (C), pH, Conductivity (mS/cm); total and free chlorine (mg/L) from municipal drinking water samples collected from homes in Martin County Kentucky between December 2018 and December 2019 (N = number of observations; SD = standard deviation).

	Season													
	Winter (Dec-Feb)			Spring (Mar-May)		Summer (Jun-Aug)			Fall (Sep-Nov)			All		
	Mean	N	SD	Mean	N	Mean	N	SD	Mean	N	SD	Mean	N	SD
**TTHMs (mg/L)**	0.030	29	0.020	0.047	1	0.072	45	0.021	0.099	21	0.027	0.065	96	0.034
**HAA5 (mg/L)**	0.020	29	0.007	0.030	1	0.049	44	0.013	0.027	21	0.010	0.035	95	0.017
**conductivity mS/cm**	0.338	29	0.195	0.311	1	0.353	45	0.060	0.755	21	0.101	0.436	96	0.210
**free Cl (mg/L)**	1.7	29	0.4	2.0	1	1.3	44	0.5	1.1	20	0.5	1.4	94	0.5
**total Cl (mg/L)**	2.0	29	0.6	1.7	1	1.4	45	0.5	1.3	20	0.5	1.6	95	0.6
**pH**	7.2	29	0.5	7.2	1	7.2	45	0.2	7.8	20	0.3	7.4	95	0.4
**Temperature °C**	8.7	29	3.3	20.0	1	24.0	45	2.1	17.9	20	4.8	18.0	95	7.3
**Distance km**	12.6	29	5.8	7.5	1	14.0	45	8.4	12.9	20	5.3	13.36	95	6.9
**Water age (hours)**	61.0	28	96.6	26.6	1	47.0	35	42.6	60.0	20	108.5	54.5	84	80.8

**Table 2.** Comparison of U.S. EPA maximum contaminant level goals (MCLG) with average concentrations detected in Martin County, KY, USA. Note that only observations above the method reporting limits were used to compute means.

Compound	MCLG (mg/L)	Martin County mean (n above reporting limit)
Chloroform	0.07	0.035 (95)
Dibromochloromethane	0.046	0.010 (90)
Bromodichloromethane	0	0.019 (95)
Bromoform	0	0.005 (32)
Dichloroacetic acid	0	0.017 (95)
Trichloroacetic acid	0.02	0.012 (94)
Monochloroacetic acid	0.07	0.003 (2)
Bromoacetic acid	NA	0.003 (6)
Dibromoacetic acid	NA	0.018 (50)

**Table 3.** Spatial autocorrelation of total trihalomethanes and haloacetic acids in water samples from Martin County, KY (Dec 2018- Dec 2019). Higher Moran’s I value indicates more spatial clustering of high values. Pesudo p-value < 0.05 indicates statistical significance.

	Total Trihalomethanes (TTHM)	Haloacetic Acids (HAA5)
All seasons (n = 97)	*I = −0.002; No clustering of similar values (pseudo p-value = 0.435)*	*I = 0.177; Some clusteringof similar values (pseudo p-value = 0.005)*
Summer (n = 42)	*I = 0.189; Some clustering of similar values (pseudo p-value = 0.015)*	*I = 0.193; Some clustering of similar values (pseudo p-value = 0.023)*
Fall (n = 30)	*I = −0.104; No clustering of similar values (pseudo p-value = 0.298)*	*I = 0.050; No clustering of similar values (pseudo p-value = 0.177)*
Winter (n = 18)	*I = 0.084; No clustering of similar values (pseudo p-value = 0.140)*	*I = 0.233; Some clustering of similar values (pseudo p-value = 0.036)*
Spring (n = 6)	*Not enough observations*	*Not enough observations*

## Data Availability

Data are available from the University of Kentucky Center for Clinical and Translational Science RedCAP data repository (https://www.ccts.uky.edu/services-resources-researchers/redcap) for researchers who meet the criteria for access to human subjects data. To access the data, please contact the database administrator, Brent Seeders (brent.seeders@uky.edu) and request access to data from the Martin County Drinking Water Project.
