# Supplementary material for: Spatial and seasonal variation in disinfection byproducts concentrations in a rural public drinking water system: A case study of Martin County, Kentucky, USA
Source: PLOS Water. Author manuscript; Available in PMC 2024 Aug 22. (PMC11340270; doi:10.1371/journal.pwat.0000227)
Supplement: S1 — Table. Multiple regression coefficients for haloacetic acids (HAA5). [file NIHMS2015761-supplement-S1.pdf]

Coefficients[a]

Model		Unstandardized Coefficients		Standardized Coefficients	t	Sig.
		B	Std. Error	Beta		
	(Constant)	-.065	.035		-1.840	.069
	conductivity	-.037	.011	-.461	-3.294	.001
	ph	.011	.005	.283	2.084	.040
	temperature	.002	.000	.690	9.853	<.001
	Distance	.001	.000	.298	4.030	<.001

a. Dependent Variable: total_haa5
