# Supplementary material for: Spatial and seasonal variation in disinfection byproducts concentrations in a rural public drinking water system: A case study of Martin County, Kentucky, USA
Source: PLOS Water. Author manuscript; Available in PMC 2024 Aug 22. (PMC11340270; doi:10.1371/journal.pwat.0000227)
Supplement: S3 — Table. Multiple regression coefficients for dichloroacetic acid. [file NIHMS2015761-supplement-S3.pdf]

Coefficients[a]

Model		Unstandardized Coefficients		Standardized Coefficients	t	Sig.
		B	Std. Error	Beta		
	(Constant)	.003	.002		1.253	.214
	temperature	.001	.000	.672	8.024	<.001
	Distance	.000	.000	.171	2.040	.045

a. Dependent Variable: Dichloroacetic_acid
