# Supplementary material for: Spatial and seasonal variation in disinfection byproducts concentrations in a rural public drinking water system: A case study of Martin County, Kentucky, USA
Source: PLOS Water. Author manuscript; Available in PMC 2024 Aug 22. (PMC11340270; doi:10.1371/journal.pwat.0000227)
Supplement: S2 — Table. Multiple regression coefficients for total triahalomethanes. [file NIHMS2015761-supplement-S2.pdf]

Coefficients[a]

Model		Unstandardized Coefficients		Standardized Coefficients	t	Sig.
		B	Std. Error	Beta		
	(Constant)	.023	.008		2.791	.006
	conductivity	.085	.007	.530	11.409	<.001
	free_chlorine	-.022	.003	-.348	-7.116	<.001
	temperature	.002	.000	.434	9.286	<.001

a. Dependent Variable: total_trihalomethanes
