# Supplementary material for: Spatial and seasonal variation in disinfection byproducts concentrations in a rural public drinking water system: A case study of Martin County, Kentucky, USA
Source: PLOS Water. Author manuscript; Available in PMC 2024 Aug 22. (PMC11340270; doi:10.1371/journal.pwat.0000227)
Supplement: S4 — Table. Multiple regression coefficients for trichloroacetic acid. [file NIHMS2015761-supplement-S4.pdf]

Coefficients[a]

Model	Unstandardized Coefficients		Standardized Coefficients	t	Sig.
	B	Std. Error	Beta		
(Constant)	-.020	.012		-1.640	.106
conductivity	-.022	.005	-.514	-4.368	<.001
free_chlorine	-.003	.001	-.283	-3.975	<.001
ph	.005	.002	.311	2.731	.008
temperature	.001	.000	.652	9.459	<.001
Distance	.000	.000	.165	2.339	.022

a. Dependent Variable: trichloroacetic_acid
