# Supplementary material for: Spatial and seasonal variation in disinfection byproducts concentrations in a rural public drinking water system: A case study of Martin County, Kentucky, USA
Source: PLOS Water. Author manuscript; Available in PMC 2024 Aug 22. (PMC11340270; doi:10.1371/journal.pwat.0000227)
Supplement: S5 — Table. Multiple regression coefficients for dibromoacetic acid. [file NIHMS2015761-supplement-S5.pdf]

Coefficients[a]

Model		Unstandardized Coefficients		Standardized Coefficients	t	Sig.
		B	Std. Error	Beta		
	(Constant)	-.004	.001		-4.716	<.001
	conductivity	.014	.002	.850	8.707	<.001

a. Dependent Variable: Dibromoacetic_acid
