# Supplementary material for: Spatial and seasonal variation in disinfection byproducts concentrations in a rural public drinking water system: A case study of Martin County, Kentucky, USA
Source: PLOS Water. Author manuscript; Available in PMC 2024 Aug 22. (PMC11340270; doi:10.1371/journal.pwat.0000227)
Supplement: S6 — Table. Multiple regression coefficients for chloroform. [file NIHMS2015761-supplement-S6.pdf]

Coefficients[a]

Model		Unstandardized Coefficients		Standardized Coefficients	t	Sig.
		B	Std. Error	Beta		
5	(Constant)	.026	.007		3.922	<.001
	free_chlorine	-.015	.003	-.362	-5.054	<.001
	temperature	.002	.000	.621	8.677	<.001

a. Dependent Variable: Chloroform
