# Supplementary material for: Spatial and seasonal variation in disinfection byproducts concentrations in a rural public drinking water system: A case study of Martin County, Kentucky, USA
Source: PLOS Water. Author manuscript; Available in PMC 2024 Aug 22. (PMC11340270; doi:10.1371/journal.pwat.0000227)
Supplement: S7 — Table. Multiple regression coefficients for dibromochloromethane. [file NIHMS2015761-supplement-S7.pdf]

Coefficients[a]

Model		Unstandardized Coefficients		Standardized Coefficients		
		B	Std. Error	Beta	t	Sig.
	(Constant)	.012	.006		2.094	.040
	conductivity	.041	.002	1.084	17.200	<.001
	total_chlorine	-.001	.000	-.098	-2.512	.015
	ph	-.003	.001	-.192	-3.049	.003
	temperature	-4.924E-5	.000	-.069	-1.770	.081

a. Dependent Variable: Dibromochloromethane
