# Supplementary material for: Spatial and seasonal variation in disinfection byproducts concentrations in a rural public drinking water system: A case study of Martin County, Kentucky, USA
Source: PLOS Water. Author manuscript; Available in PMC 2024 Aug 22. (PMC11340270; doi:10.1371/journal.pwat.0000227)
Supplement: S8 — Table. Multiple regression coefficients for bromodichloromethane. [file NIHMS2015761-supplement-S8.pdf]

Coefficients[a]

Model		Unstandardized Coefficients B	Unstandardized Coefficients Std. Error	Standardized Coefficients Beta	t	Sig.
3	(Constant)	-.001	.002		-.666	.508
	conductivity	.036	.002	.710	16.588	<.001
	free_chlorine	-.003	.001	-.192	-4.211	<.001
	temperature	.000	.000	.406	9.213	<.001
	Distance	.000	.000	.085	1.932	.057

a. Dependent Variable: bromodichloromethane
