# Supplementary figures and images for: Spatial and seasonal variation in disinfection byproducts concentrations in a rural public drinking water system: A case study of Martin County, Kentucky, USA
Source: PLOS Water. Author manuscript; Available in PMC 2024 Aug 22. (PMC11340270; doi:10.1371/journal.pwat.0000227)

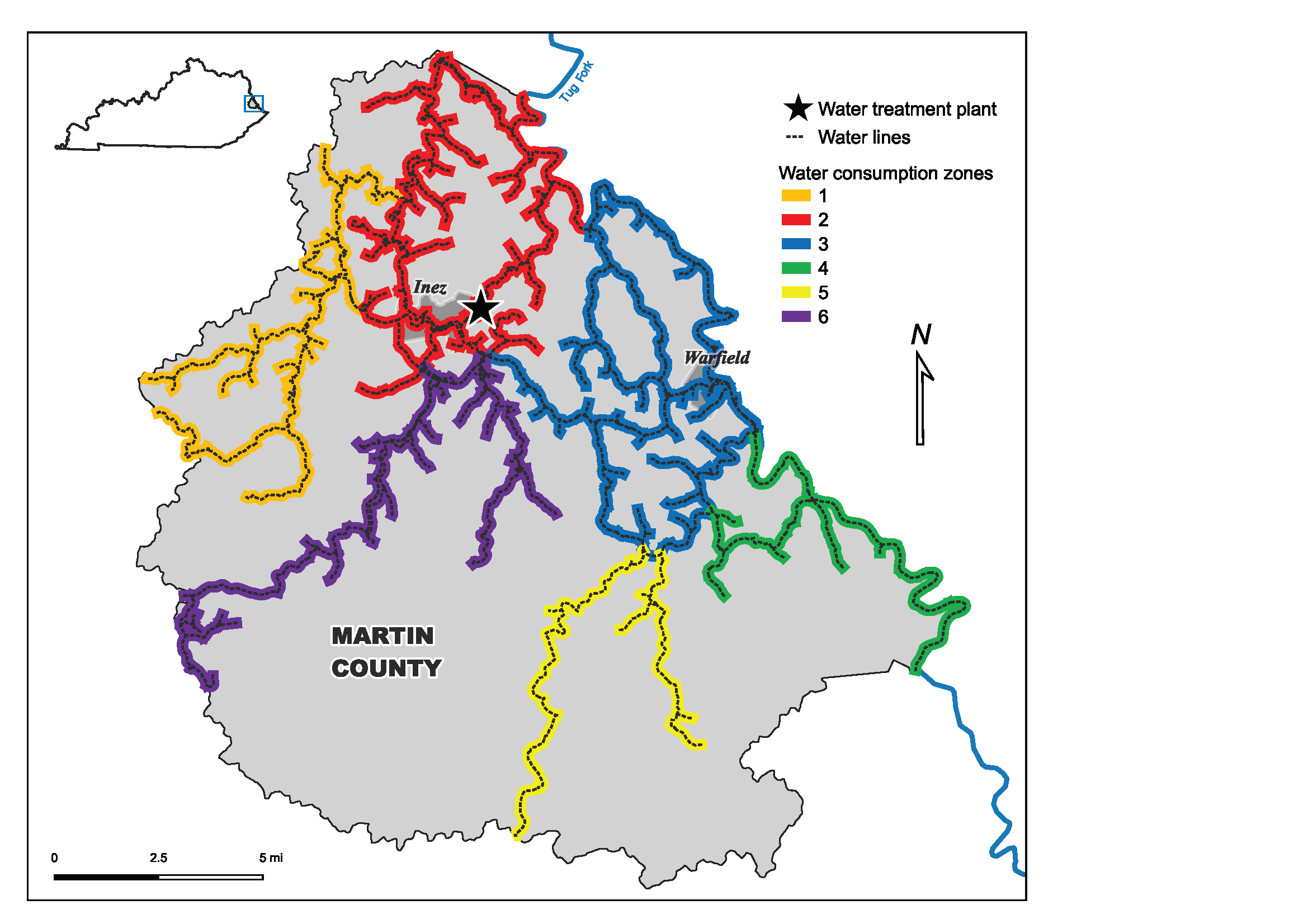

Supplement: S9 — S1 Fig. Water consumption zones used for multiple regression analysis for the Martin County Water District labelled using different colors as indicated in the key. [file NIHMS2015761-supplement-S9.tiff]

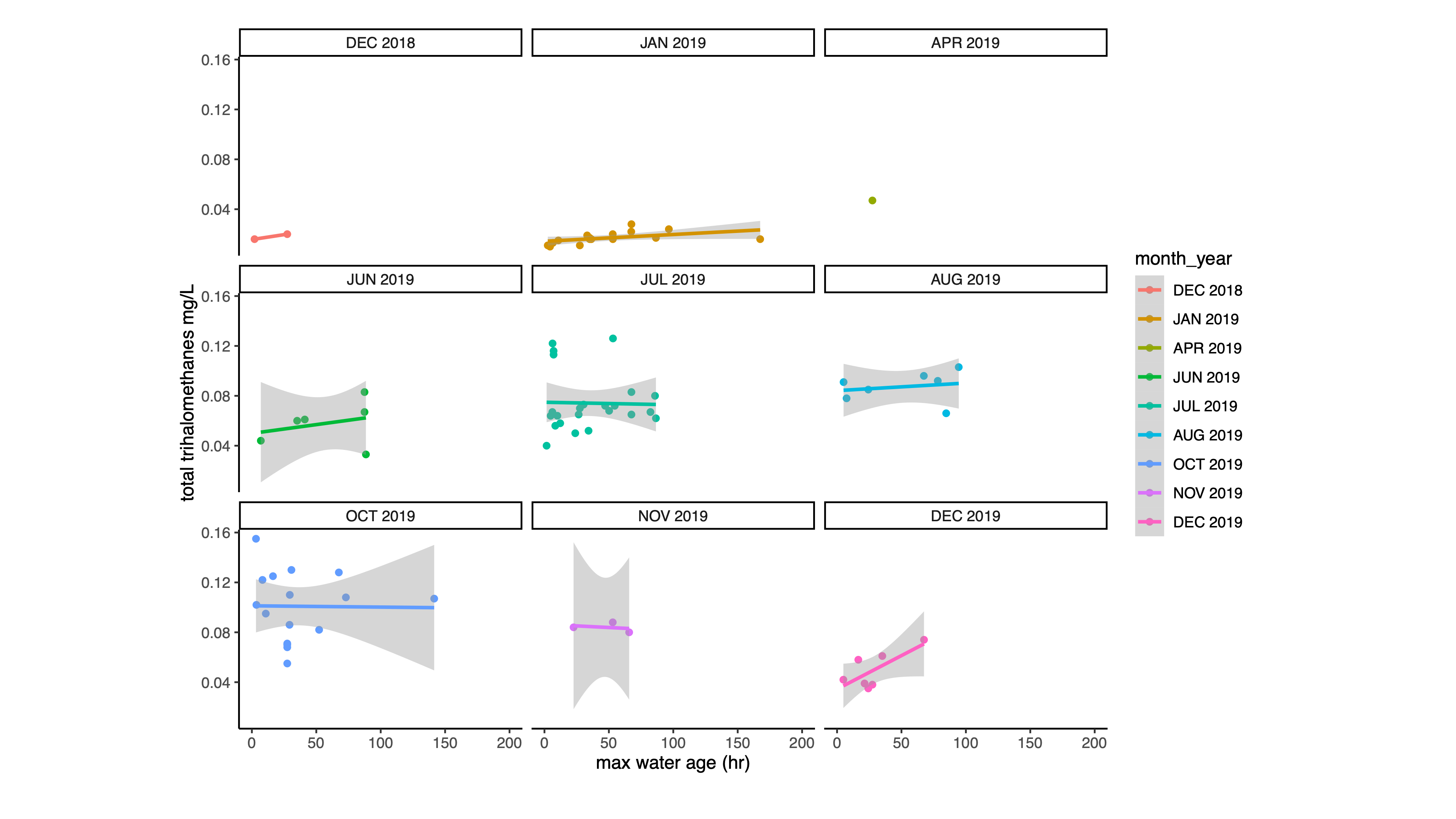

Supplement: S10 — S2 Fig. Total trihalomethanes plotted as a function of estimated maximum water age by month. Solid line is the least squares fit of the data and the shaded areas represent the 95% confidence interval for the fitted model. We obtained the GIS data comprising the county boundary polygon data for Fig 3 and, thus, the base layer for these maps—from the Kentucky Geography Network (https://kygeonet.ky.gov), the spatial data clearinghouse for Kentucky. A ZIP file containing this publicly available shapefile can be downloaded from https://ky.app.box.com/v/kymartian-KyBndsCounty/folder/137608414025. We used state cartographic boundary files from the U.S. Census for the inset U.S. map (https://www.census.gov/geographies/mapping-files/timeseries/geo/carto-boundary-file.2015.html), and the National Hydrography Data (NHD) from the United States Geological Survey (USGS; https://www.usgs.gov/national-hydrography/national-hydrography-dataset) for the Tug Fork River. [file NIHMS2015761-supplement-S10.tiff]

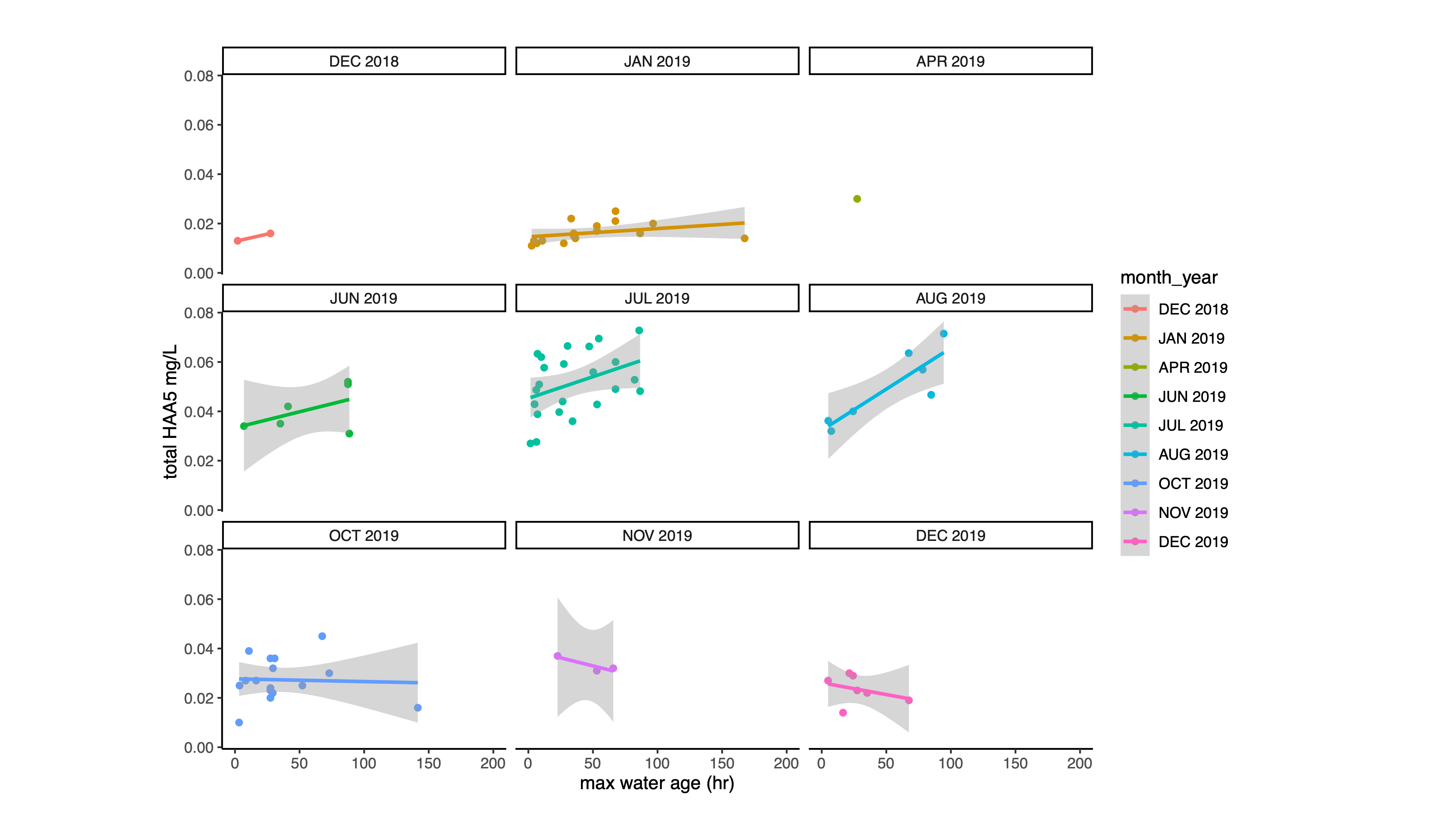

Supplement: S11 — S3 Fig. Total haloacetic acids (HAA5) plotted as a function of estimated maximum water age by month. Solid line is the least squares fit of the data and the shaded areas represent the 95% confidence interval for the fitted model. [file NIHMS2015761-supplement-S11.tiff]

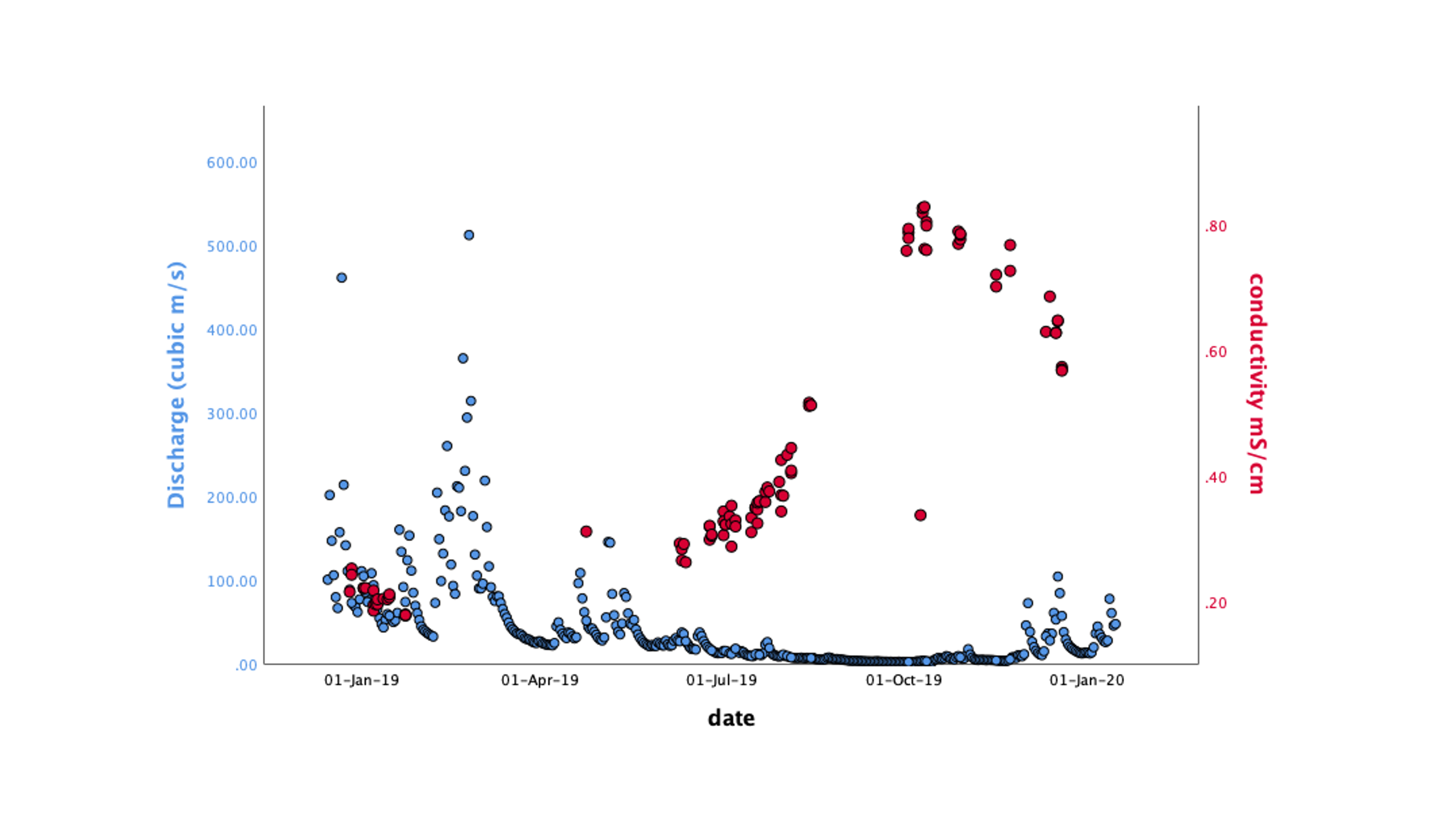

Supplement: S12 — S4 Fig. Relationship between discharge of the Tug Fork River at Williamson, WV and conductivity of drinking water in Martin County, KY. [file NIHMS2015761-supplement-S12.tiff]

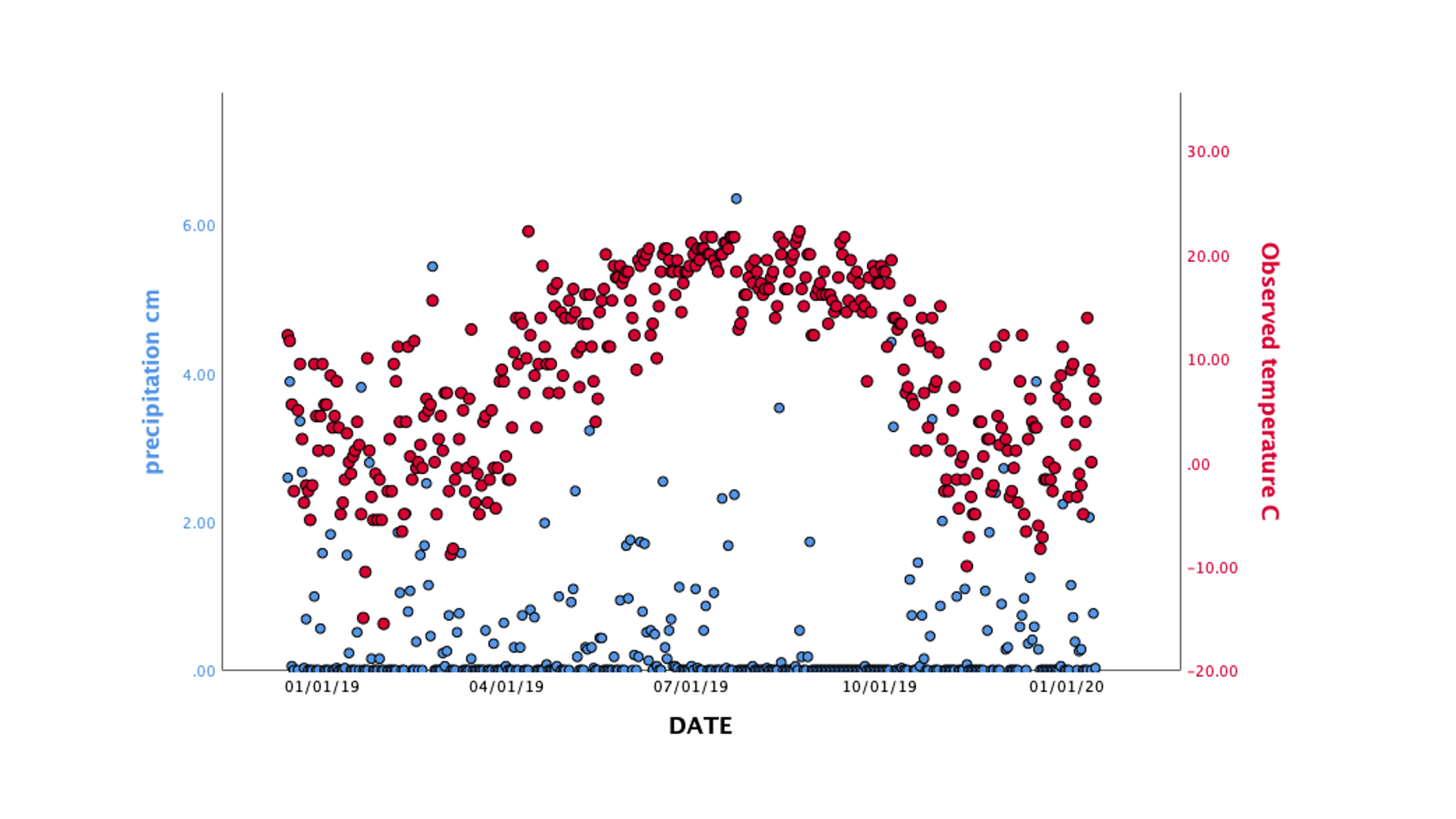

Supplement: S13 — S5 Fig. Precipitation and observed air temperature at Martin County, KY from December 2018 to January 2020. [file NIHMS2015761-supplement-S13.tiff]

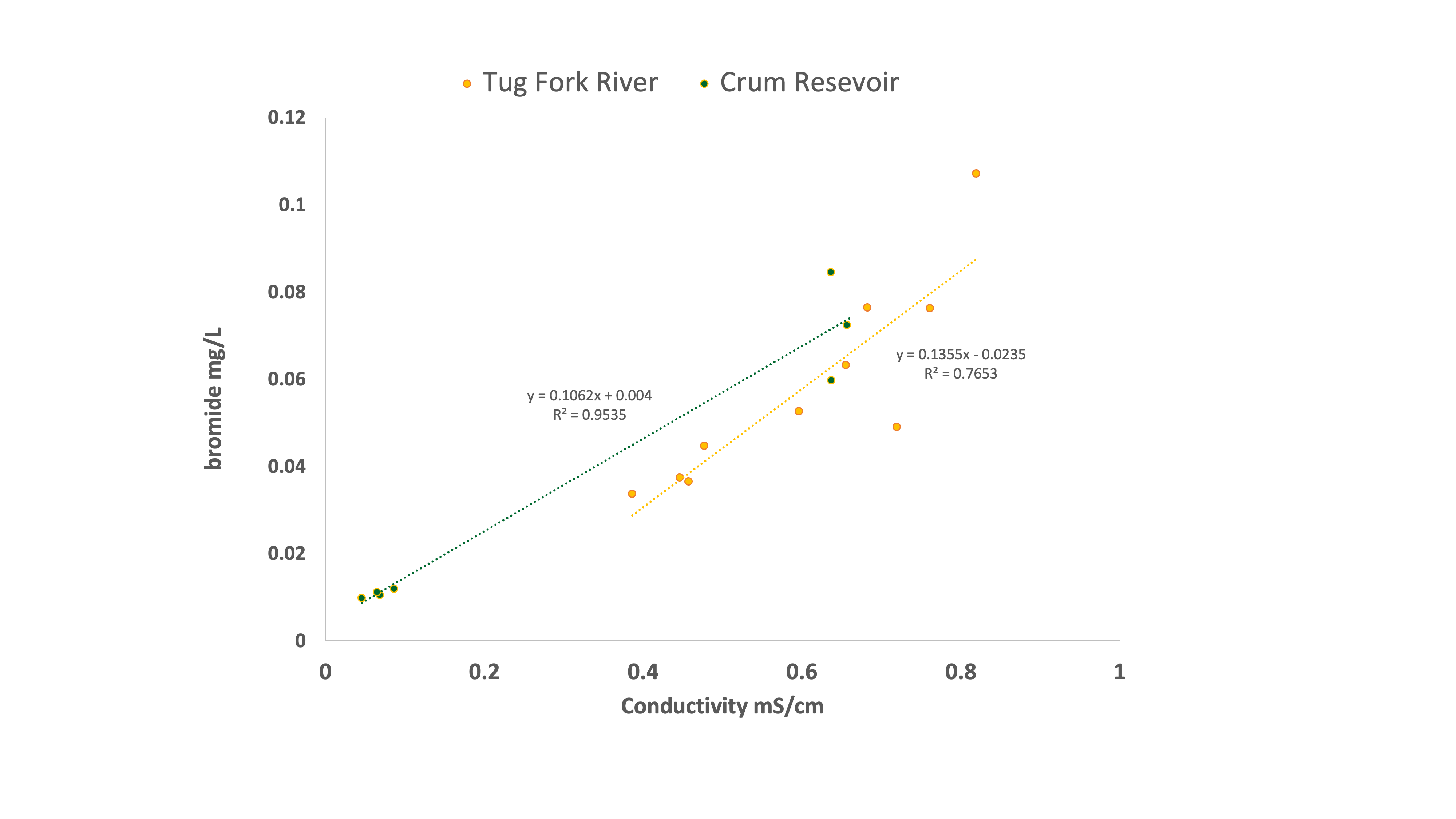

Supplement: S14 — S6 Fig. Relationship between conductivity and bromide concentrations in the Tug Fork River at Kermit West Virginia, USA and the Curtis Crum Reservoir, Martin County, KY USA. Samples collected between September 2021 and May 2022. [file NIHMS2015761-supplement-S14.tiff]
